# Machine Learning–Based Prediction of In-Hospital Falls in Adult Inpatients: Retrospective Observational Multicenter Study

**DOI:** 10.2196/75958

**Published:** 2025-12-04

**Authors:** Takuya Nishino, Kotone Matsuyama, Yasuo Miyagi, Nari Tanabe, Fumiko Yamaguchi, Hiroki Ito, Shizuka Soh, Ayako Yano, Masako Mizuno, Katsuhito Kato, Hiroshige Jinnouchi, Chol Kim, Yosuke Ishii, Hiroki Yamaguchi, Yukihiro Kondo

**Affiliations:** 1 Department of Health Policy and Management Nippon Medical School Tokyo Japan; 2 Center for Clinical Research and Development National Center for Child Health and Development Tokyo Japan; 3 Department of Medical Safety Control Nippon Medical School Hospital Tokyo Japan; 4 Department of Cardiovascular Surgery Nippon Medical School Tokyo Japan; 5 Faculty of Engineering Suwa University of Science Nagano Japan; 6 Department of Medical Safety Control Nippon Medical School, Chiba Hokusoh Hospital Chiba Japan; 7 Nursing Department Nippon Medical School, Chiba Hokusoh Hospital Chiba Japan; 8 Department of Hygiene and Public Health Nippon Medical School Tokyo Japan; 9 Department of Anesthesiology Nippon Medical School, Chiba Hokusoh Hospital Chiba Japan; 10 Department of Hematology Nippon Medical School Tokyo Japan; 11 Department of Urology Nippon Medical School Tokyo Japan

**Keywords:** falls, machine learning, predictive model, inpatients, risk assessment, hospitals, calibration

## Abstract

**Background:**

Falls among hospitalized patients are a critical issue that often leads to prolonged hospital stays and increased health care costs. Traditional fall risk assessments typically rely on standardized scoring systems; however, these may fail to capture the complex and multifactorial nature of fall risk factors.

**Objective:**

This retrospective observational multicenter study aimed to develop and validate a machine learning–based model to predict in-hospital falls and to evaluate its performance in terms of discrimination and calibration.

**Methods:**

We analyzed the data of 83,917 inpatients aged 65 years and older with a hospital stay of at least 3 days. Using Diagnosis Procedure Combination data and laboratory results, we extracted demographic, clinical, functional, and pharmacological variables. Following the selection of 30 key features, 4 predictive models were constructed: logistic regression, extreme gradient boosting, light gradient boosting machine (LGBM), and categorical boosting (CatBoost). The synthetic minority oversampling technique and isotonic regression calibration were applied to improve the prediction quality and address class imbalance.

**Results:**

Falls occurred in 2173 (2.6%) patients. CatBoost achieved the highest *F*_1_-score (0.189, 95% CI 0.162-0.215) and area under the precision-recall curve (0.112, 95% CI 0.091-0.136), whereas LGBM had the best calibration slope (0.964, 95% CI 0.858-1.070) with good discrimination (*F*_1_-score 0.182, 95% CI 0.156-0.209; area under the precision-recall curve 0.094, 95% CI 0.078-0.113). Logistic regression had the lowest discrimination (*F*_1_-score 0.120, 95% CI 0.100-0.143). Shapley Additive Explanations analysis consistently identified low albumin, impaired transfer ability, and the use of sedative-hypnotics or diabetes medications as major contributors to fall risk. In incident report analysis (n=435), 49.2% of falls were toileting-related, peaking between 4 and 6 AM, with bedside falls predominating in high or very high risk groups.

**Conclusions:**

CatBoost and LGBM offer clinically valuable prediction performance, with CatBoost favored for high-risk patient identification and LGBM for probability-based intervention thresholds. Integrating such models into electronic health records could enable real-time risk scoring and trigger targeted interventions (eg, toileting assistance and mobility support). Future work should incorporate dynamic, time-varying patient data to improve real-time risk prediction.

## Introduction

Falls among hospitalized patients represent a considerable concern that can adversely affect patient health outcomes and the quality of care. Falls are often associated with prolonged hospital stays and increased hospitalization costs [1]. The World Health Organization defines a fall as “an event that results in a person coming to rest inadvertently on the ground, floor, or other lower level, excluding intentional position changes to lean against furniture, walls, or other objects” [2]. Several studies have identified various risk factors associated with falls, including advanced age, a history of falls, reduced activities of daily living (ADL), the use of sleeping medications, and malnutrition [3-6]. Given the multifactorial and complex nature of fall risk, numerous guidelines for fall prevention in hospitalized patients have been established worldwide [7-9]. These guidelines recommend performing multifactorial assessments for each patient rather than relying solely on standardized fall risk scores. Similar to many other hospitals in Japan, at our institution, we adopt a scoring system based on a number of risk factors. This scoring system facilitates easy evaluation and achieves high sensitivity [10]. However, applying a uniform evaluation to all patients may limit specificity and fail to reflect varying clinical contexts; as a result, individual clinical differences and interactions among risk factors may be overlooked [11]. This limitation underscores the multifaceted and complex nature of fall-related risk.

In Japan, nurses primarily assess fall risk through bedside clinical observation. However, limited time for patient care and insufficient staffing of registered nurses are associated with higher fall rates, underscoring the need for effective prediction models [12]. To capture these complex risk factors more accurately, recent studies have increasingly focused on machine learning–based models for fall prediction models [13]. These models are capable of analyzing complex interactions among numerous variables and can identify patterns that traditional scoring systems may overlook. Previous studies conducted in Japanese university hospitals have primarily evaluated the performance of these models using the area under the receiver operating characteristic curve (ROAUC) and reported values between 0.78 and 0.90 [14-17]. However, in clinical practice, calibration accuracy is as important as discriminatory performance. Poor calibration may lead to excessive interventions for patients classified as high risk, increasing the workload of medical staff and health care costs. Conversely, insufficient care for patients classified as low risk could result in severe fall incidents, highlighting the critical need for calibration evaluation. Despite its importance, reports on the calibration performance of machine learning models remain limited.

In this study, we aimed to develop a fall prediction model for hospitalized patients and validate its performance using discriminatory and calibration metrics. Additionally, we aimed to elucidate the factors that contribute to falls to support the development and implementation of more effective fall-prevention strategies.

## Methods

### Study Design and Data Collection

This retrospective observational multicenter study was conducted using inpatient data from 2 affiliated hospitals of Nippon Medical School: Nippon Medical School Hospital and Chiba Hokusoh Hospital. The dataset was compiled from the Diagnosis Procedure Combination data and laboratory test results extracted from electronic medical records.

### Ethical Considerations

Ethics approval for this study was obtained from the central ethics review committee of Nippon Medical School (approval M-2023-137). This study adhered to the principles of the Declaration of Helsinki. Informed consent was obtained using an opt-out approach, and the study was reported following the TRIPOD (Transparent Reporting of a Multivariable Prediction Model for Individual Prognosis or Diagnosis) guidelines [18]. No compensation was provided to the participants because this study was a retrospective observational study using de-identified data.

### Patients and Outcomes

All the patients included in this study were hospitalized between April 2018 and March 2023. Patients younger than 65 years of age and those with a hospital stay of less than 3 days were excluded. Patient and feature selection were guided by a fall risk assessment sheet used at the study facility. As the assessment sheet identified individuals aged 65 years or older as a fall risk factor, patients younger than 65 years of age were excluded accordingly (Table S1 in Multimedia Appendix 1). The outcome was defined as the first occurrence of a fall within 30 days of the admission date based on incident reports. These reports are considered highly reliable due to mandatory reporting requirements, and they contain detailed information such as the time, location, trigger of the fall, and classification into mild or severe cases.

### Variables

The Diagnosis Procedure Combination data contained information on all hospitalized patients, including demographic details (such as age and sex), physical metrics (height, weight, and BMI, calculated by dividing body weight in kilograms by the square of height in meters), and clinical information (comorbidities, procedures, medications, and consciousness levels at admission) [19]. Medications were defined according to those included in the hospital fall risk assessment protocol. The physical condition of the patients was assessed using the nursing care needs score on admission [19]. This score measures the level of assistance required for activities such as turning over, transferring, oral hygiene, eating, and changing clothes (0 points=no assistance, 1 point=partial assistance, and 2 points=full assistance). It also evaluated one’s ability to understand medical instructions (0 points=yes and 1 point=no) and the presence of risky behaviors (0 points=no and 2 points=yes; Table S2 in Multimedia Appendix 1).

Blood test results were obtained from electronic medical records to assess known factors influencing falls, such as anemia, malnutrition, and electrolyte imbalance. The test results were defined as the closest sample collected to the admission date within 60 days prior to admission. To minimize missing values, we extracted blood test results and medication-related variables 60 days before the admission date, rather than strictly limiting extraction to the admission date. Before model development, we assessed multicollinearity among all candidate predictors by calculating the variance inflation factor (VIF). All variables had VIF values <5, indicating no concerning multicollinearity. Therefore, all variables were retained for model construction without further dimensionality reduction (Table S3 in Multimedia Appendix 1).

### Imputation of Missing Data

Missing BMI values and blood test results were imputed using a single imputation in the Python *MissForest* package [20,21]. As the missing rate for all variables was below 20%, all variables were included in the analysis. A 20% threshold was used as the exclusion criterion.

### Predictive Model Development

The dataset was randomly divided into 80% training and 20% test sets using stratified sampling to ensure that the end-point occurrence rates were preserved, as in the original population. We used extreme gradient boosting (XGB) to select the top 30 features based on their importance in the training data [22]. These selected features were used for subsequent modeling and analysis. The following predictive models were used: conventional logistic regression (LR), XGB, light gradient boosting machine (LGBM), and categorical boosting (CatBoost) [23,24]. To address class imbalance, we applied the synthetic minority oversampling technique algorithm to artificially increase the proportion of fall cases to 20% of the total cases in the training data [25]. Additionally, class-balanced loss functions were used during the training phase to mitigate the effects of imbalanced data. Hyperparameter optimization for each model was performed using Optuna for up to 500 trials (Table S4 in Multimedia Appendix 1) [26]. The average precision score was used as the evaluation metric. To improve model calibration, we used isotonic regression to adjust the predicted probabilities of each model [27]. Finally, all models were trained using a 10-fold stratified k-fold cross-validation.

### Model Interpretation

Shapley Additive Explanations (SHAP) values were used to interpret the output of the machine learning models [28]. SHAP is a game-theoretic approach to explain individual predictions by calculating each feature’s marginal contribution to the difference between the actual prediction and the average prediction across the dataset. SHAP values provide quantitative insights into the contribution (positive or negative) of each feature to the predicted fall risk for a given patient, thereby enhancing the interpretability and transparency of the decision-making process of the model.

### Model Validation

The optimal threshold for each model was determined by maximizing the *F*_1_-score to achieve the highest possible predictive performance. Then, calibration was applied to ensure that the predicted probabilities accurately reflected the true probabilities. Additionally, the bootstrap method was applied to the test set to evaluate the performance of the predictive models. A total of 3000 bootstrap resamples obtained from the test set were used to calculate 95% CIs for each performance metric. The discriminatory ability of the model was assessed using the ROAUC and area under the precision-recall curve (PRAUC), a threshold-independent metric that quantifies the average precision across all possible recall values, providing a robust measure of the ability of a model to correctly identify positive cases, particularly in imbalanced datasets. The predicted probabilities for the calibration plots were divided into 10 percentiles, and the mean predicted and observed probabilities of the outcome for each bin were plotted. Two indices were used to evaluate the calibration: the calibration slope, which indicates the agreement between the predicted probabilities and the observed outcomes; and the Brier score, which measures the accuracy of the probability predictions. A calibration slope closer to 1 and a Brier score closer to 0 indicate ideal model performance.

### Statistical Analysis

All statistical analyses were performed using Python (version 3.10.10; Python Software Foundation).

## Results

### Study Population and Baseline Characteristics

**Figure 1:**
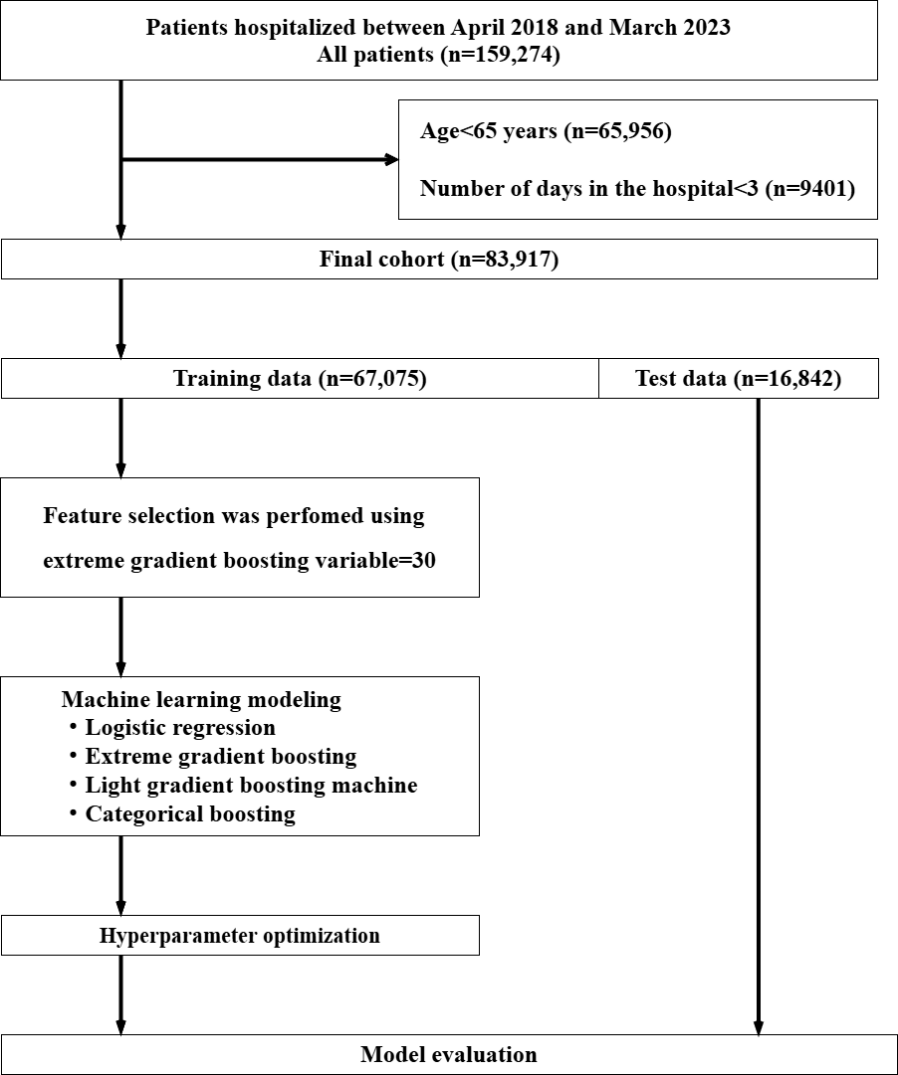
Flowchart for patient assignment and model development.

Among the initial 159,274 patients, 65,956 were younger than 65 years of age, and 9401 had a hospital stay of less than 3 days. Therefore, 83,917 participants were included in the final analysis after applying the exclusion criteria. Of these, 67,705 (80%) were allocated to the training dataset and 16,842 (20%) to the validation dataset (Figure 1). Table 1 presents the key variables included in the final prediction models, along with their descriptive statistics in the overall, training, and test datasets. Complete descriptive statistics for all candidate variables prior to feature selection, including VIF values, are provided in Table S3 in Multimedia Appendix 1. Outcomes were observed in 2173 (2.6%) patients, and the characteristics of the training and test datasets were well balanced. The 30 selected features used in the analysis are illustrated in Multimedia Appendix 2. Multimedia Appendix 2 highlights the relative importance of each variable, with risky behavior (0.148), oral hygiene (0.086), and unconsciousness (Japan Coma Scale≠0; 0.044) among the top predictors, providing context for the feature selection process.

**Table 1:** Characteristics of patients included in the predictive model.

Variable		Overall (N=83,917)		Training dataset (n=67,075)	Test dataset (n=16,842)
Outcome, n (%)		2173 (2.6)		1733 (2.6)	440 (2.6)
**Demographic**					
	Age (years), median (IQR)		75 (71-81)	75 (71-81)	75 (71-81)
	Male sex, n (%)		50,623 (60.3)	40,435 (60.3)	10,188 (60.5)
	Emergency admission, n (%)		31,819 (37.9)	25,491 (38)	6328 (37.6)
	Unconsciousness (JCS^a^≠0), n (%)		10,925 (13)	8796 (13.1)	2129 (12.6)
	Intensive care unit on admission, n (%)		9655 (11.5)	7716 (11.5)	1939 (11.5)
**Transfer, n (%)^b^**					
	0		61,528 (73.3)	49,135 (73.3)	12,393 (73.6)
	1		16,066 (19.1)	12,884 (19.2)	3182 (18.9)
	2		6323 (7.5)	5056 (7.5)	1267 (7.5)
**Food intake, n (%)^b^**					
	0		69,759 (83.1)	55,721 (83.1)	14,038 (83.4)
	1		9955 (11.9)	7973 (11.9)	1982 (11.8)
	2		4203 (5)	3381 (5)	822 (4.9)
**Dressing or undressing, n (%)^b^**					
	0		62,796 (74.8)	50,199 (74.8)	12,597 (74.8)
	1		12,427 (14.8)	9947 (14.8)	2480 (14.7)
	2		8694 (10.4)	6929 (10.3)	1765 (10.5)
Oral hygiene, n (%)^b^		19,923 (23.7)		15,946 (23.8)	3977 (23.6)
Ability to understand medical instructions, n (%)^b^		8604 (10.3)		6876 (10.3)	1728 (10.3)
Risky behavior, n (%)^b^		5599 (6.7)		4511 (6.7)	1088 (6.5)
Nursing care needs score, median (IQR)		0 (0-3)		0 (0-3)	0 (0-3)
**Comorbidity, n (%)**					
	Certain infectious diseases (A00-B99)		7257 (8.6)	5814 (8.7)	1443 (8.6)
	Neoplasms (C00-D48)		29,679 (35.4)	23,637 (35.2)	6042 (35.9)
	Diseases of the blood (D50-D89)		13,214 (15.7)	10,583 (15.8)	2631 (15.6)
	Endocrine diseases (E00-E90)		37,836 (45.1)	30,302 (45.2)	7534 (44.7)
	Mental disorders (F00-F99)		5878 (7)	4721 (7)	1157 (6.9)
	Nervous system diseases (G00-G99)		20,222 (24.1)	16,249 (24.2)	3973 (23.6)
	Respiratory diseases (J00-J99)		15,862 (18.9)	12,705 (18.9)	3157 (18.7)
	Digestive diseases (K00-K93)		46,192 (55)	36,981 (55.1)	9211 (54.7)
	Skin and subcutaneous tissue diseases (L00-L99)		5741 (6.8)	4533 (6.8)	1208 (7.2)
	Injury and poisoning (S00-T98)		14,297 (17)	11,471 (17.1)	2826 (16.8)
	Special purpose codes (U00-U99)		1041 (1.2)	857 (1.3)	184 (1.1)
**Medications at admission**					
	Medication score, median (IQR)		3 (2-4)	3 (2-4)	3 (2-4)
	Antiparkinsonism drugs, n (%)		1579 (1.9)	1264 (1.9)	315 (1.9)
	Hypnotics and sedatives, n (%)		20,146 (24)	16,048 (23.9)	4098 (24.3)
	Chemotherapy agents, n (%)		10,926 (13)	8686 (12.9)	2240 (13.3)
	Antiplatelet or coagulation drugs, n (%)		29,453 (35.1)	23,545 (35.1)	5908 (35.1)
	Diabetes treatments, n (%)		19,839 (23.6)	15,858 (23.6)	3981 (23.6)
	Antihypertensive drugs, n (%)		46,430 (55.3)	37,075 (55.3)	9355 (55.5)
**Laboratory findings at admission (g/dL), median (IQR)**					
	Albumin level		3.9 (3.5-4.2)	3.9 (3.5-4.2)	3.9 (3.5-4.2)

^a^JCS: Japan Coma Scale.

^b^Nursing care needs score: 0=no assistance, 1=partial assistance, and 2=full assistance.

### Evaluation of Prediction Models

**Figure 2:**
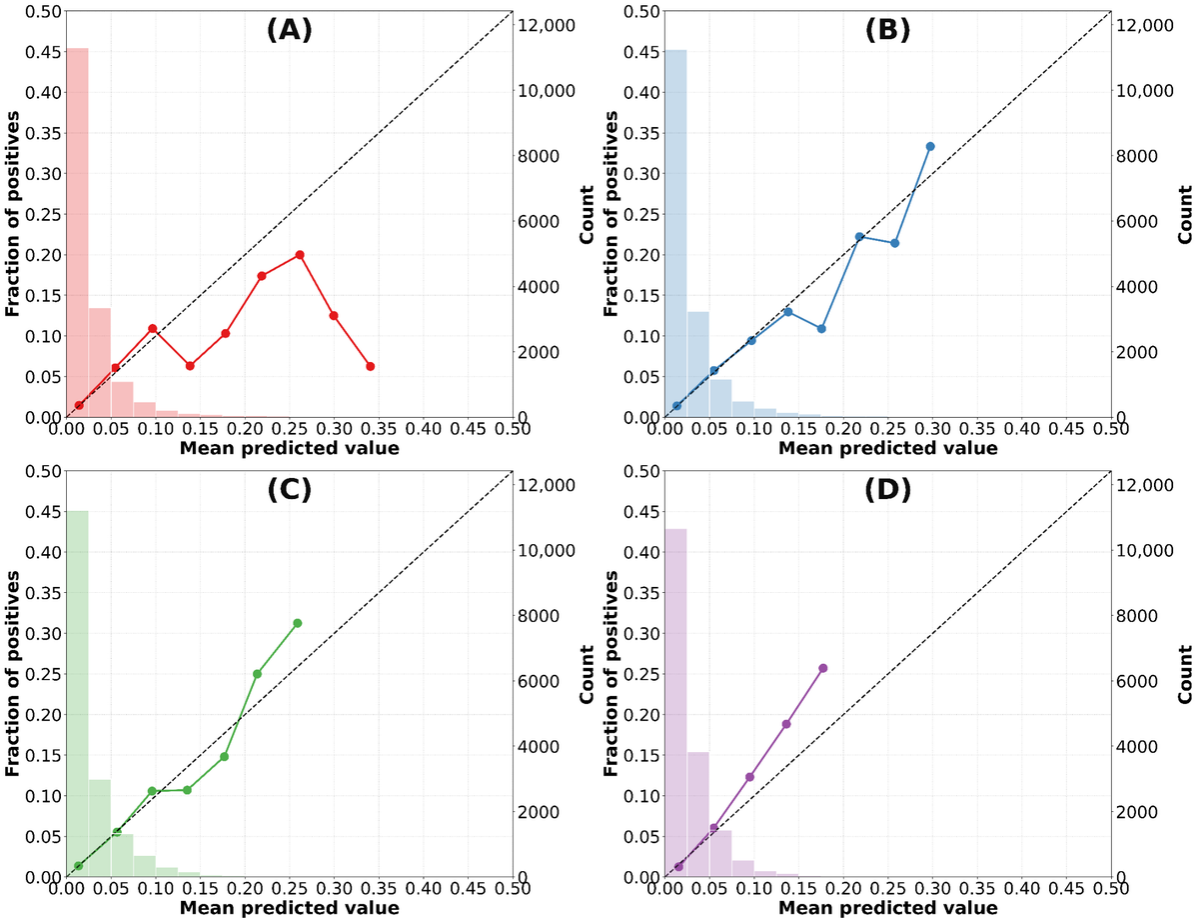
Model validations. Calibration plot of the models. (A) Logistic regression, (B) extreme gradient boosting, (C) light gradient boosting machine, and (D) categorical boosting.

We evaluated the performances of 4 classification models: LR, XGB, LGBM, and CatBoost. The performance of each model was assessed using the *F*_1_-score, PRAUC, calibration slope, and optimal threshold, with a 95% CI provided for each metric. The LR model achieved an *F*_1_-score of 0.120 (95% CI 0.100-0.143), with an optimal threshold of 0.069. Its PRAUC was 0.066 (95% CI 0.055-0.0792), and the calibration slope was 0.838 (95% CI 0.739-0.934). In comparison, XGB showed a slightly higher *F*_1_-score of 0.182 (95% CI 0.157-0.207), with an optimal threshold of 0.074. It achieved a PRAUC of 0.098 (95% CI 0.081-0.117) and had an improved calibration slope of 0.937 (95% CI 0.829-1.040). Similarly, the LGBM model demonstrated an *F*_1_-score of 0.182 (95% CI 0.156-0.209) and an optimal threshold of 0.0846. Its PRAUC was 0.094 (95% CI 0.078-0.113), and it demonstrated the most accurate calibration among all models at 0.964 (95% CI 0.858-1.070). Among the 4 models, the CatBoost model achieved the highest *F*_1_-score of 0.189 (95% CI 0.162-0.215) and an optimal threshold of 0.0724. It also recorded the higher PRAUC at 0.112 (95% CI 0.091-0.136). Although its calibration slope of 1.120 (95% CI 1.000-1.240) was slightly above 1, it was close to the ideal value. While CatBoost outperformed the other models in terms of the *F*_1_-score and PRAUC, the LGBM model demonstrated the most accurate calibration, with a calibration slope closest to 1, indicating superior reliability in risk prediction relative to the observed outcomes (Table 2 and Figure 2). The predicted probabilities of in-hospital falls were stratified assuming actual clinical use and compared with the observed in-hospital fall rates. The observed fall rates in the 4 defined risk categories (low, medium, high, and very high) fell within the range of predicted probabilities (Table 3).

**Table 2:** Model evaluation index.

Model	*F*_1_-score (95% CI)	Optimal threshold	ROAUC^a^ (95% CI)	PRAUC^b^ (95% CI)	Calibration slope (95% CI)	Brier score (95% CI)
Logistic regression	0.120 (0.100-0.143)	0.069	0.729 (0.705-0.753)	0.066 (0.055-0.0792)	0.838 (0.739-0.934)	0.025 (0.023-0.027)
Extreme gradient boosting	0.182 (0.157-0.207)	0.074	0.774 (0.751-0.798)	0.098 (0.081-0.117)	0.937 (0.829-1.040)	0.024 (0.022-0.026)
Light gradient boosting machine	0.182 (0.156-0.209)	0.085	0.774 (0.751-0.797)	0.094 (0.078-0.113)	0.964 (0.858-1.070)	0.024 (0.022-0.026)
Categorical boosting	0.189 (0.162-0.215)	0.072	0.787 (0.764-0.809)	0.112 (0.091-0.136)	1.120 (1.000-1.240)	0.024 (0.022-0.026)

^a^ROAUC: area under the receiver operating characteristic curve.

^b^PRAUC: area under the precision-recall curve.

**Table 3:** Observed outcomes for each risk stratum classified by predicted probability.

Risk stratum (range of predictive probability)			Patients (total), n (%)		Patients (outcomes), n (%)		Observed outcome rate
**Logistic regression**							
	Low: <0.02	10,064 (59.8)		96 (1)		0.01	
	Middle: ≥0.02, <0.05	4570 (27.2)		146 (3.2)		0.03	
	High: ≥0.05, <0.10	1563 (9.3)		127 (8.1)		0.08	
	Very high: ≥0.10	587 (3.5)		66 (11.2)		0.11	
**Extreme gradient boosting**							
	Low: <0.02	10,255 (60.9)		97 (0.9)		0.01	
	Middle: ≥0.02, <0.05	4289 (25.5)		134 (3.1)		0.03	
	High: ≥0.05, <0.10	1588 (9.4)		117 (7.4)		0.07	
	Very high: ≥0.10	652 (3.9)		87 (13.3)		0.13	
**Light gradient boosting machine**							
	Low: <0.02	10,375 (53.6)		91 (0.9)		0.01	
	Middle: ≥0.02, <0.05	4125 (34.1)		124 (3)		0.03	
	High: ≥0.05, <0.10	1602 (10.2)		126 (8.3)		0.08	
	Very high: ≥0.10	682 (1.5)		94 (15.4)		0.14	
**Categorical boosting**							
	Low: <0.02	9045		79		0.01	
	Middle: ≥0.02, <0.05	5753		173		0.03	
	High: ≥0.05, <0.10	1727		143		0.08	
	Very high: ≥0.10	259		40		0.15	

### Model Interpretation

**Figure 3:**
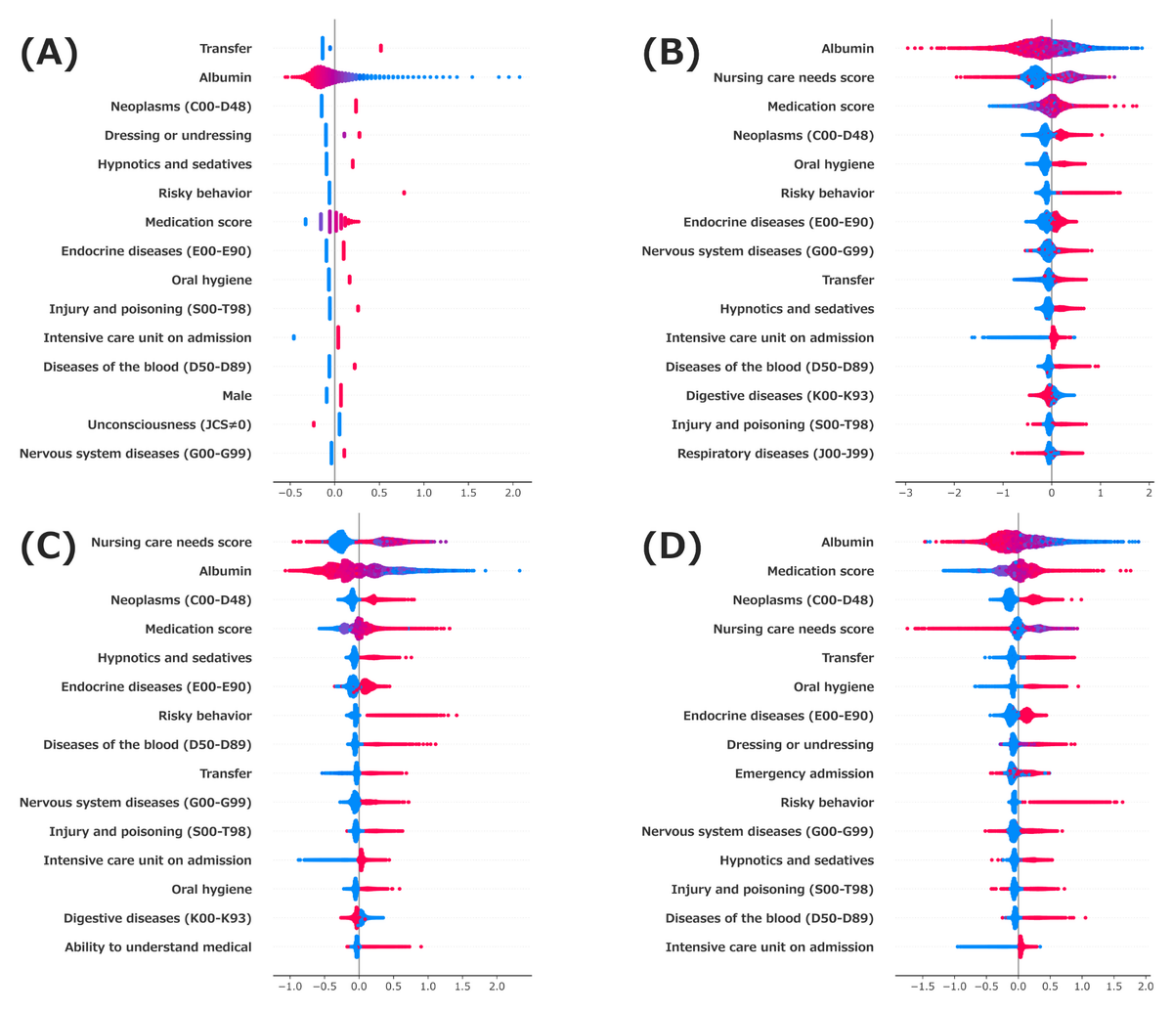
Shapley Additive Explanations values in the models. The top 20 variables of SHAP analysis in each model are shown with an impact of contribution to the prediction. (A) Logistic regression, (B) extreme gradient boosting, (C) light gradient boosting machine, and (D) categorical boosting. JCS: Japan Coma Scale.

The contribution of each feature to the prediction of outcomes was visualized using the SHAP algorithm. The features were ranked in descending order of importance, with red indicating high influence and blue indicating low influence. All 4 models identified albumin levels, transfer, and neoplasms as key predictors, although the magnitude of their impact varied across the models (Figure 3). Additionally, medications—particularly hypnotics, sedatives, and diabetes treatments—were identified as important features in all models. These findings suggest that, in addition to medical conditions, functional abilities, such as transfer and dressing or undressing, play a significant role in predicting outcomes.

### Classification From Incident Reports

We presented descriptive statistics of falls during hospitalization based on 435 incidents from the test data, categorized into different risk groups (low, middle, high, and very high) according to the predicted probabilities from the LGBM model (Table 4). These findings suggest that falls occur primarily at bedside, with the urge to defecate identified as the most common trigger. Notably, the characteristics of falls vary across risk groups; the proportion of bedside falls was higher in the high and very high risk groups (76/126, 60.3% vs 54/91, 59.3%, respectively), whereas falls occurring inside the patient room are more common in the low and middle risk groups (38/105, 36.2% and 41/113, 36.3%, respectively) These findings indicate that appropriately managing toileting needs and movements around the bedside is essential to prevent falls during hospitalization.

**Table 4:** Location and occasion of falls by risk.

		Overall (n=435)	Low (n=91)	Middle (n=124)	High (n=126)	Very high (n=94)
Occurrence date of falls during hospitalization, median (IQR)		9 (4-17)	8 (4-14)	11 (5-16)	7 (3-15)	9 (6-18)
**Location of occurrence, n (%)**						
	Bedside	218 (50.1)	32 (35.2)	56 (45.2)	76 (60.3)	54 (57.4)
	Inside the patient room	80 (18.4)	23 (25.3)	24 (19.4)	17 (13.5)	16 (17)
	Inside the toilet	57 (13.1)	10 (11)	21 (16.9)	15 (11.9)	11 (11.7)
	In the bathroom or washbasin area	15 (3.4)	5 (5.5)	5 (4)	3 (2.4)	2 (2.1)
	Inside the hospital	63 (14.5)	21 (23.1)	17 (13.7)	15 (11.9)	10 (10.6)
	Outside the hospital	2 (0.5)	0 (0)	1 (0.8)	0 (0)	1 (1.1)
**Trigger of occurrence, n (%)**						
	Urge to defecate	214 (49.2)	39 (42.9)	58 (46.8)	64 (50.8)	53 (56.4)
	Attempting to grab something	51 (11.7)	14 (15.4)	14 (11.3)	12 (9.5)	11 (11.7)
	Standing or sitting position	79 (18.2)	14 (15.4)	22 (17.7)	31 (24.6)	12 (12.8)
	Changing clothes	12 (2.8)	3 (3.3)	5 (4)	3 (2.4)	1 (1.1)
	While walking	46 (10.6)	15 (16.5)	16 (12.9)	5 (4)	10 (10.6)
	Others	33 (7.6)	6 (6.6)	9 (7.3)	11 (8.7)	7 (7.4)

Given that excretion-related falls accounted for the largest proportion of falls (217/435, 49.9%), we conducted a more detailed analysis of their characteristics.

**Figure 4:**
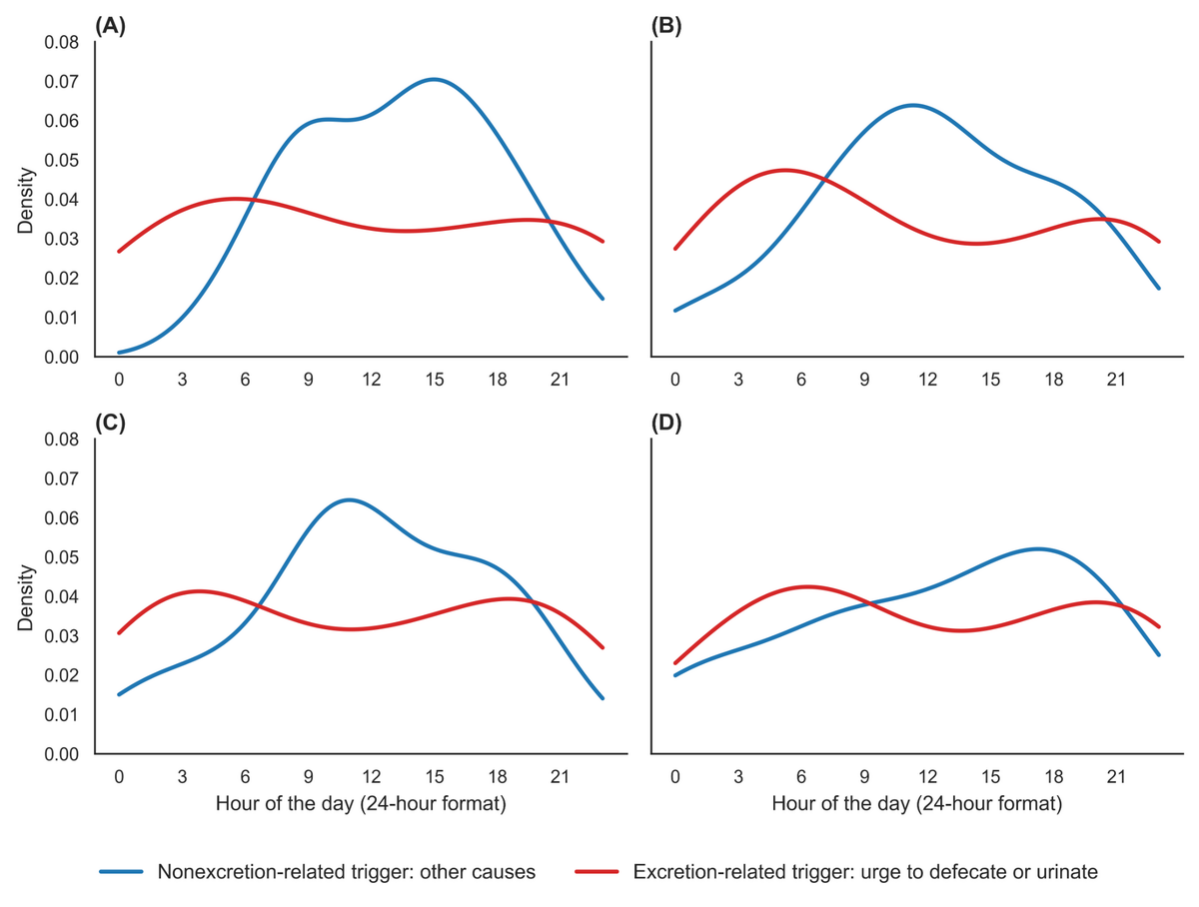
Factors in falls related to excretion. Kernel density estimation graphs showing the hourly distribution of triggers categorized by light gradient boosting machine–predicted probabilities ((A) low, (B) middle, (C) high, and (D) very high). The blue lines represent nonexcretion-related triggers (eg, other causes), and the red lines represent excretion-related triggers (urge to defecate or urinate). Density is plotted against the hour of the day (24-hour format).

Figure 4 presents the kernel density estimation of fall triggers by hour of the day, distinguishing between excretion-related triggers (such as the urge to defecate or urinate) and nonexcretion-related causes. The red curve indicates that excretion-related falls peaked in the early morning, particularly between 4 and 6 AM. In contrast, the blue curve representing nonexcretion-related falls displayed a broader temporal distribution, with a higher incidence in the afternoon. Among the 217 incidents involving toileting, 132 (61%) occurred while moving to the toilet, 52 (24%) occurred inside the toilet, and 33 (15%) occurred while returning from the toilet.

## Discussion

### Principal Findings

This study evaluated the predictive accuracy and interpretability of machine learning models for assessing the in-hospital fall risk. Using LR, XGB, LGBM, and CatBoost, we aimed to identify high-risk patients and categorize falls based on their backgrounds and functional abilities. Our findings revealed that CatBoost achieved the highest *F*_1_-score and PRAUC, whereas LGBM demonstrated the best calibration slope, closely approximating the ideal for risk prediction. Notably, functional abilities, such as transfer, and clinical factors, such as albumin and neoplasms, consistently contributed to fall prediction across the models. Further analysis of incident reports revealed that fall locations and triggers varied by risk level; falls near the bedside were more frequent in higher-risk groups, whereas excretion-related falls often occurred in the early morning hours. These findings suggest that proactive management of toileting and bedside movements may effectively reduce the incidence of falls among hospitalized patients.

In previous studies on machine learning–based fall prediction models, the reported ROAUC ranged from 0.7 to 0.9 [13]. Similarly, this study demonstrated a comparable predictive performance. However, it is important to note that model performance is influenced by several factors, including algorithms used, techniques for data balancing, data augmentation, and methods for handling missing values. To address class imbalance in this study, we applied the synthetic minority oversampling technique algorithm to increase the proportion of fall cases from 2.6% to 20%. While this approach improved model training, it may have altered the underlying data distribution and could lead to overestimation of predicted probabilities for the minority class (fall incidents). Therefore, such artificial balancing should be interpreted with caution, as it may introduce inherent bias into model performance metrics and clinical applicability. In comparing the models in this study, boosting algorithms exhibited a slight advantage over LR, likely due to their ability to capture the complexity of fall predictions more effectively. Although the existing literature on calibration is limited, the event incidence observed in each risk layer fell within the predicted probability ranges (low, middle, high, and very high), suggesting that appropriate risk stratification for falls was achieved. When evaluating the model utility, poor calibration can lead to overestimation or underestimation of risk, which poses substantial concern [29]. In clinical settings, overestimating fall risk could lead to inefficient allocation of nursing resources, potentially diverting attention from patients truly at high risk, whereas underestimating risk may delay preventive interventions and increase adverse outcomes. Therefore, calibration is particularly important when predicted probabilities are linked to intervention thresholds or staffing decisions. The LGBM model developed in this study demonstrated reasonable calibration, as shown in the calibration plot, effectively mitigating the risk of overestimation and underestimation. Among the 4 models evaluated in this study, the boosting models exhibited good performance. Specifically, CatBoost proved more effective for discrimination, whereas LGBM was most suitable for calibration. In clinical application, the choice between models should depend on the intended use. Miscalibration may result in inefficient allocation of nursing resources or delayed preventive interventions, underscoring the importance of calibration when predicted probabilities are directly linked to intervention thresholds or staffing decisions. For scenarios where intervention thresholds are defined by predicted probabilities, a well-calibrated model such as LGBM may be preferable. In contrast, when resources are limited and identifying the highest-risk patients is the priority, CatBoost may be more advantageous. Future studies could also explore ensemble approaches that combine both strengths. Practical integration of the model into the electronic health record could allow nurses to view individualized fall risk scores in real time, and predefined thresholds could trigger tailored preventive interventions (eg, increased observation, assistance with toileting, or targeted rehabilitation exercises). Future prospective studies are needed to determine the optimal threshold settings and evaluate their impact on clinical outcomes. However, research on more precise calibration algorithms that use neural networks is ongoing, and the application of these methods could offer further improvements [30].

The key features identified in this study include decreased serum albumin levels, limitations in ADL, and the use of sedative-hypnotic medications, all of which are consistent with findings of previous reports [31]. Additionally, changes in gait, impaired balance, history of falls, muscle weakness, and polypharmacy have been identified as significant factors contributing to falls. These factors overlap with certain abnormalities in locomotive frailty, highlighting the importance of comprehensive assessment of nutritional status and ADL [32]. Importantly, these high-impact features are clinically modifiable to varying degrees. For example, low albumin levels may be addressed through nutritional supplementation, protein-enriched diets, and early mobilization programs. Impaired ADL, particularly transfer ability, could be targeted with physiotherapy and environmental adjustments to reduce mobility-related hazards. For patients receiving sedative-hypnotics or antidiabetic drugs, careful medication review, dose adjustment, or substitution should be considered to minimize adverse effects (eg, dizziness or hypoglycemia). Integrating these targeted interventions into clinical workflows could help translate model outputs into personalized fall-prevention strategies. From a pharmacological perspective, sedative medications and diabetes treatments have been identified as significant contributors to fall risk. Several studies have consistently reported that antipsychotics, antidepressants, and benzodiazepines are associated with a high risk of falls across [4]. Moreover, hypoglycemia and hyperglycemia during diabetes treatment increase the risk of falls in hospitalized patients. Abnormal blood glucose levels (either <70 mg/dL or >200 mg/dL) are associated with an increased risk of falls, and treatment with insulin or oral hypoglycemic agents is considered a contributing factor [33]. Patients with diabetes often present multiple comorbidities that can increase the risk of polypharmacy, further increasing the likelihood of falls [34,35]. These findings underscore the need for a comprehensive approach to reduce the risk of falls.

Toileting-related falls accounted for a significant percentage of the incidents (49.2%), particularly during the night. Nighttime toileting has been reported to be a significant risk factor, especially when associated with intravenous fluid infusion, history of falls, visual impairment, and gait instability [36]. The high frequency of falls occurring during transfer from beds or chairs to toilets supports these findings [37]. It has also been noted that the urge to urinate can increase walking speed and stride length, thereby heightening the risk of falls. This effect is closely related to the severity of urinary incontinence, and gait is strongly associated with complex cognitive functions. As cognitive function declines, executive function is also impaired, which can lead to abnormal gait patterns [38]. Additionally, the use of sleeping medications should be considered another factor contributing to toileting-related falls. These sleeping medications can impair reaction speed upon awakening in older adults, potentially affecting balance and muscle control [39,40]. Benzodiazepines and other sedatives have been shown to cause dizziness during nighttime awakening, further increasing the risk of falling during toilet-related activities. Therefore, it is essential to assess the use of sleeping medications and consider alternative drugs or dose reductions when necessary.

### Limitations

This study has some limitations. First, as a retrospective study, the explanatory variables for the machine learning models were limited to those that could be easily collected from clinical data and had minimal missing values. Consequently, data such as fall history, vital signs, social factors, and implementation of fall-prevention measures were excluded. Second, because the study was conducted solely in medical facilities in Japan, the findings may not be generalizable to patients from other regions. Differences in health care delivery systems, patient demographics, and cultural practices related to fall prevention could influence the baseline risk and the effectiveness of prediction models. Therefore, future studies should validate the machine learning models using independent external data from hospitals in diverse geographic regions and health care systems, ideally in settings with different staffing models, resource availability, and fall-prevention protocols. Third, this study used only 4 machine learning algorithms (LR, XGB, LGBM, and CatBoost), whereas other algorithms, such as neural networks and support vector machines, may demonstrate superior predictive performance. Finally, the features used were limited to those available at the time of admission, and dynamic changes during hospitalization (eg, postoperative status, new-onset infections, and medication adjustments) were not considered. The absence of time-dependent variables, including repeated assessments of vital signs, mobility, nutritional status, and mental state, may limit the ability of the model to capture short-term fluctuations in fall risk. Incorporating such dynamic patient data and continuous risk assessment throughout hospitalization could enable the development of models that more accurately reflect real-time risk and support timely, individualized preventive interventions. Future research integrating these factors, along with advanced transformer-based model architectures capable of capturing complex temporal and contextual relationships, may lead to more accurate and clinically useful prediction models [41].

Additionally, as with any machine learning approach, there is a potential risk of algorithmic bias, which may arise if certain patient subgroups (eg, older individuals, those with cognitive impairment, or those from resource-limited backgrounds) are underrepresented or systematically differ in predictor distributions. Such biases could inadvertently lead to unequal predictive performance across subpopulations, potentially exacerbating disparities in fall prevention efforts. Thus, future studies should include fairness assessment and bias mitigation strategies to ensure that prediction models are equitable and beneficial across all patient groups.

### Conclusions

We developed a machine learning–based model for predicting inpatient fall risk and assessed its performance in terms of discrimination and calibration, achieving excellent calibration. SHAP analysis identified several factors associated with an increased risk of falls, including indicators of frailty and the use of sedatives, hypnotics, and diabetes medications. A detailed analysis of fall incidents revealed that toileting-related falls—particularly those occurring near the bedside during late-night and early morning hours—were prevalent. These findings underscore the importance of implementing preventive measures tailored to individual patient conditions and behaviors, such as mitigating risk during toilet transfers and maintaining balance upon awakening. We believe that these insights contribute to the development of more precise and effective fall-prevention strategies in hospitals.

## Appendix

Multimedia Appendix 1 The falls assessment tool, severity and nursing care needs scoring, baseline characteristics and missing data of the study population, and hyperparameter settings for machine learning models.

Multimedia Appendix 2 Top 30 most important features selected for model development.

## Abbreviations

ADL: activities of daily living
CatBoost: categorical boosting
LGBM: light gradient boosting machine
LR: logistic regression
PRAUC: area under the precision-recall curve
ROAUC: area under the receiver operating characteristic curve
SHAP: Shapley Additive Explanations
TRIPOD: Transparent Reporting of a Multivariable Prediction Model for Individual Prognosis or Diagnosis
VIF: variance inflation factor
XGB: extreme gradient boosting

## Data Availability

The datasets generated and analyzed during this study are not publicly available due to ethical restrictions and patient confidentiality concerns, as they contain sensitive information derived from Diagnosis Procedure Combination data and electronic medical records. However, deidentified data may be available from the corresponding author upon reasonable academic request and subject to approval by the institutional ethics committee.
