# Supplementary material for: Machine Learning–Based Prediction of In-Hospital Falls in Adult Inpatients: Retrospective Observational Multicenter Study
Source: JMIR Med Inform. 2025 Dec 4;13:e75958. doi: 10.2196/75958 (PMC12715471; doi:10.2196/75958)
Supplement: Multimedia Appendix 1 [file medinform_v13i1e75958_app1.docx]

**Table S1.** **Falls and falls assessment tool.**

| Category | Variable |
| --- | --- |
| A. Age | Aged 65 years or older |
| B. Medical History | History of falls or slips |
| C. Sensory | Vision impairment |
|  | Hearing impairment |
|  | Balance impairment |
| D. Motor Function | Paralysis or numbness |
|  | Weakness in legs or reduced muscle strength |
| E. Activity Level | Unsteadiness |
|  | Can walk freely |
|  | Uses a wheelchair, cane, or walker |
|  | Requires assistance for mobility |
|  | Bedridden but can move limbs |
| F. Cognitive Function | Cognitive impairment |
|  | Agitation |
|  | Reduced judgment, comprehension, or memory |
|  | Disorientation or consciousness disturbance |
| G. Medication | Hypnotics or sedatives |
|  | Antipyretic analgesics |
|  | Narcotics |
|  | Laxatives |
|  | Antihypertensives or diuretics |
|  | Anxiolytics |
|  | Anticancer drugs |
|  | Antiplatelet or anticoagulant agents |
|  | Antiparkinsonian drugs |
|  | Antipsychotic drugs |
| H. Excretion | Urinary or fecal incontinence |
|  | Frequent urination |
|  | Frequent nighttime toilet use |
|  | Requires assistance for toileting |
|  | Uses a portable toilet |

**Table S2. Severity and nursing care needs.**

|  | 0 points | 1 point | 2 points |
| --- | --- | --- | --- |
| Turning over in bed | Can do | With some help | Cannot do |
| Transfer | No assistance | Partial assistance | Full assistance |
| Oral hygiene | No assistance | Needs assistance |  |
| Food intake | No assistance | Partial assistance | Full assistance |
| Dressing/undressing | No assistance | Partial assistance | Full assistance |
| Ability to understand medical instructions /nursing instructions | Yes | No |  |
| Risky behavior | No |  | Yes |

**Table S3. Baseline characteristics, missing data, and variance inflation factors (VIFs) for all candidate predictors used in model development.**

| Variable | Overall | Training dataset | Test dataset | Missing cases, n (%) | VIF ^b^ |
| --- | --- | --- | --- | --- | --- |
|  | 83,917 | 67,075 | 16,842 |  |  |
| Outcomes, n (%) | 2173 ( 2.6) | 1733 ( 2.6) | 440 ( 2.6) | 0 (0.0) |  |
| Demographics |  |  |  |  |  |
| Age (years) | 75 (71-81) | 75 (71-81) | 75 (71-81) | 0 (0.0) | 1.26 |
| Male sex, n (%) | 50623 (60.3) | 40435 (60.3) | 10188 (60.5) | 0 (0.0) | 1.17 |
| Body mass index (kg/m^2^) | 22.5 (20.3-24.9) | 22.5 (20.3-24.9) | 22.5 (20.2-24.9) | 3,097 (3.8) | 1.15 |
| Emergency admission, n (%) | 31819 (37.9) | 25491 (38.0) | 6328 (37.6) | 0 (0.0) | 1.83 |
| Unconsciousness (JCS≠0), n (%) ^c^ | 10925 (13.0) | 8796 (13.1) | 2129 (12.6) | 0 (0.0) | 1.75 |
| Intensive Care Unit on admission,n (%) | 9655 (11.5) | 7716 (11.5) | 1939 (11.5) | 0 (0.0) | 1.73 |
| Dialysis, n (%) | 3910 ( 4.7) | 3128 ( 4.7) | 782 ( 4.6) | 0 (0.0) | 2.23 |
| Turning over in bed, n (%) ^d^ |  |  |  |  | 2.49 |
| 0 | 60488 (72.1) | 48318 (72.0) | 12170 (72.3) | 0 (0.0) |  |
| 1 | 14059 (16.8) | 11272 (16.8) | 2787 (16.5) | 0 (0.0) |  |
| 2 | 9370 (11.2) | 7485 (11.2) | 1885 (11.2) | 0 (0.0) |  |
| Transfer, n (%) ^d^ |  |  |  |  | 1.52 |
| 0 | 61528 (73.3) | 49135 (73.3) | 12393 (73.6) | 0 (0.0) |  |
| 1 | 16066 (19.1) | 12884 (19.2) | 3182 (18.9) | 0 (0.0) |  |
| 2 | 6323 ( 7.5) | 5056 ( 7.5) | 1267 ( 7.5) | 0 (0.0) |  |
| Food intake, n (%) ^d^ |  |  |  |  | 2.08 |
| 0 | 69759 (83.1) | 55721 (83.1) | 14038 (83.4) | 0 (0.0) |  |
| 1 | 9955 (11.9) | 7973 (11.9) | 1982 (11.8) | 0 (0.0) |  |
| 2 | 4203 ( 5.0) | 3381 ( 5.0) | 822 ( 4.9) | 0 (0.0) |  |
| Dressing/ Undressing, n (%) ^d^ |  |  |  |  | 3.04 |
| 0 | 62796 (74.8) | 50199 (74.8) | 12597 (74.8) | 0 (0.0) |  |
| 1 | 12427 (14.8) | 9947 (14.8) | 2480 (14.7) | 0 (0.0) |  |
| 2 | 8694 (10.4) | 6929 (10.3) | 1765 (10.5) | 0 (0.0) |  |
| Oral hygiene, n (%) ^d^ | 19923 (23.7) | 15946 (23.8) | 3977 (23.6) | 0 (0.0) | 3.02 |
| Ability to understand medical, n (%) ^d^ | 8604 (10.3) | 6876 (10.3) | 1728 (10.3) | 0 (0.0) | 2.27 |
| Risky behavior, n (%) ^d^ | 5599 ( 6.7) | 4511 ( 6.7) | 1088 ( 6.5) | 0 (0.0) | 1.88 |
| Nursing care needs score | 0 (0-3) | 0 (0-3) | 0 (0-3) | 0 (0.0) |  |
| Comorbidities |  |  |  |  |  |
| Certain infectious diseases(A00-B99), n (%) | 7257 ( 8.6) | 5814 ( 8.7) | 1443 ( 8.6) | 0 (0.0) | 1.11 |
| Neoplasms (C00-D48), n (%) | 29679 (35.4) | 23637 (35.2) | 6042 (35.9) | 0 (0.0) | 1.48 |
| Diseases of the blood (D50-D89), n (%) | 13214 (15.7) | 10583 (15.8) | 2631 (15.6) | 0 (0.0) | 1.33 |
| Endocrine diseases (E00-E90), n (%) | 37836 (45.1) | 30302 (45.2) | 7534 (44.7) | 0 (0.0) | 1.36 |
| Mental disorders (F00-F99), n (%) | 5878 ( 7.0) | 4721 ( 7.0) | 1157 ( 6.9) | 0 (0.0) | 1.22 |
| Nervous system diseases (G00-G99), n (%) | 20222 (24.1) | 16249 (24.2) | 3973 (23.6) | 0 (0.0) | 1.24 |
| Eye diseases (H00-H59), n (%) | 4205 ( 5.0) | 3381 ( 5.0) | 824 ( 4.9) | 0 (0.0) | 1.01 |
| Diseases of the ear (H60-H95), n (%) | 603 ( 0.7) | 499 ( 0.7) | 104 ( 0.6) | 0 (0.0) | 1.01 |
| Circulatory diseases (I00-I99), n (%) | 44435 (53.0) | 35485 (52.9) | 8950 (53.1) | 0 (0.0) | 1.55 |
| Respiratory diseases (J00-J99), n (%) | 15862 (18.9) | 12705 (18.9) | 3157 (18.7) | 0 (0.0) | 1.13 |
| Digestive diseases (K00-K93), n (%) | 46192 (55.0) | 36981 (55.1) | 9211 (54.7) | 0 (0.0) | 1.18 |
| Skin and subcutaneous tissue diseases (L00-L99), n (%) | 5741 ( 6.8) | 4533 ( 6.8) | 1208 ( 7.2) | 0 (0.0) | 1.03 |
| Musculoskeletal diseases (M00-M99), n (%) | 17171 (20.5) | 13667 (20.4) | 3504 (20.8) | 0 (0.0) | 1.15 |
| Genitourinary diseases (N00-N99), n (%) | 13919 (16.6) | 11096 (16.5) | 2823 (16.8) | 0 (0.0) | 1.40 |
| Pregnancy and congenital malformations (O00-Q99), n (%) | 360 ( 0.4) | 282 ( 0.4) | 78 ( 0.5) | 0 (0.0) | 1.00 |
| Injury and poisoning (S00-T98), n (%) | 14297 (17.0) | 11471 (17.1) | 2826 (16.8) | 0 (0.0) | 1.10 |
| Special Purpose Codes (U00-U99), n (%) | 1041 ( 1.2) | 857 ( 1.3) | 184 ( 1.1) | 0 (0.0) | 1.03 |
| Medications at admission |  |  |  |  |  |
| Medication Score | 3 (2-4) | 3 (2-4) | 3 (2-4) | 0 (0.0) |  |
| Steroid, n (%) | 12025 (14.3) | 9559 (14.3) | 2466 (14.6) | 0 (0.0) | 1.21 |
| Laxatives / Enemas, n (%) | 26801 (31.9) | 21373 (31.9) | 5428 (32.2) | 0 (0.0) | 1.13 |
| Diuretics, n (%) | 20471 (24.4) | 16349 (24.4) | 4122 (24.5) | 0 (0.0) | 1.27 |
| Antiepileptic Drugs, n (%) | 2795 ( 3.3) | 2264 ( 3.4) | 531 ( 3.2) | 0 (0.0) | 1.09 |
| Antiparkinsonism Drugs, n (%) | 1579 ( 1.9) | 1264 ( 1.9) | 315 ( 1.9) | 0 (0.0) | 1.05 |
| Hypnotics and sedatives, n (%) | 20146 (24.0) | 16048 (23.9) | 4098 (24.3) | 0 (0.0) | 1.15 |
| Anti psychiatric Drugs, n (%) | 6351 ( 7.6) | 5090 ( 7.6) | 1261 ( 7.5) | 0 (0.0) | 1.21 |
| Chemotherapy Agents, n (%) | 10926 (13.0) | 8686 (12.9) | 2240 (13.3) | 0 (0.0) | 1.46 |
| Antiplatelet / Coagulation Drugs, n (%) | 29453 (35.1) | 23545 (35.1) | 5908 (35.1) | 0 (0.0) | 1.40 |
| Diabetes treatments, n (%) | 19839 (23.6) | 15858 (23.6) | 3981 (23.6) | 0 (0.0) | 1.24 |
| Analgesic Drugs, n (%) | 32704 (39.0) | 26053 (38.8) | 6651 (39.5) | 0 (0.0) | 1.15 |
| Antihypertensive Drugs, n (%) | 46430 (55.3) | 37075 (55.3) | 9355 (55.5) | 0 (0.0) | 1.27 |
| Opioid Analgesics, n (%) | 5785 ( 6.9) | 4596 ( 6.9) | 1189 ( 7.1) | 0 (0.0) | 1.22 |
| Laboratory findings at admission |  |  |  |  |  |
| Aspartate transaminase (IU/L) | 18 (13-26) | 18 (13-26) | 18 (13-26) | 1994 (2.4) | 2.59 |
| Alanine transaminase (IU/L) | 23 (18-31) | 23 (18-31) | 23 (18-31) | 1991 (2.4) | 2.60 |
| Albumin (g/dL) | 3.9 (3.5-4.2) | 3.9 (3.5-4.2) | 3.9 (3.5-4.2) | 5420 (6.9) | 1.95 |
| Blood urea nitrogen (mg/dL) | 18.0 (14.2-23.5) | 18.0 (14.2-23.5) | 18.0 (14.2-23.7) | 1961 (2.4) | 2.32 |
| Creatine kinase (IU/L) | 86 (55-132) | 86 (55-132) | 86 (56-131) | 4240 (5.3) | 1.04 |
| Creatinine (mg/dL) | 0.87 (0.69-1.13) | 0.87 (0.69-1.13) | 0.87 (0.69-1.13) | 1981 (2.4) | 3.22 |
| Hemoglobin (g/dL) | 12.7 (11.1-14.0) | 12.7 (11.1-14.0) | 12.7 (11.1-14.0) | 1979 (2.4) | 1.94 |
| Potassium (mEq/L) | 4.2 (3.9-4.5) | 4.2 (3.9-4.5) | 4.2 (3.9-4.5) | 2088 (2.6) | 1.24 |
| Sodium (mEq/L) | 141 (138-142) | 141 (138-142) | 141 (138-142) | 2105 (2.6) | 1.22 |
| White blood cells (103/µL) | 6.4 (5.1-8.3) | 6.4 (5.1-8.3) | 6.3 (5.1-8.3) | 1979 (2.4) | 1.11 |

ᵃ Continuous variables are presented as median (interquartile range); categorical variables as number (%).
ᵇ VIF: variance inflation factor. Values >5 generally indicate high multicollinearity.
ᶜ JCS: Japan Coma Scale.
ᵈ Nursing care needs score item: 0 = no assistance, 1 = partial assistance, 2 = full assistance.

**Table S4. Hyperparameter search ranges and selected values for machine learning models.**

| Model | Hyperparameter | Search Range | Selected Value |
| --- | --- | --- | --- |
| Logistic Regression | penalty | ['l1', 'l2', 'elasticnet'] | l1 |
|  | C | loguniform(1e-4, 1e2) | 0.736 |
|  | l1_ratio | float(0.0, 1.0) if penalty == "elasticnet" else N/A | None |
|  | class_weight | [None, 'balanced'] | None |
| Extreme gradient boosting | max_depth | int(3, 15) | 9.0 |
|  | learning_rate | loguniform(1e-4, 1e-1) | 0.055 |
|  | subsample | float(0.5, 1.0) | 0.935 |
|  | colsample_bytree | float(0.5, 1.0) | 0.538 |
|  | reg_alpha | loguniform(1e-4, 1e2) | 0.827 |
|  | reg_lambda | loguniform(1e-4, 1e2) | 52.479 |
|  | min_child_weight | int(1, 20) | 9.000 |
|  | gamma | loguniform(1e-4, 1e2) | 0.000 |
| Light gradient boosting machine | max_depth | int(3, 15) | 10.000 |
|  | learning_rate | loguniform(1e-4, 1e-1) | 0.032 |
|  | num_leaves | int(31, 255) | 150.0 |
|  | subsample | float(0.5, 1.0) | 0.867 |
|  | colsample_bytree | float(0.5, 1.0) | 0.509 |
|  | reg_alpha | loguniform(1e-4, 1e2) | 0.001 |
|  | reg_lambda | loguniform(1e-4, 1e2) | 35.427 |
|  | min_child_samples | int(5, 100) | 20.0 |
| Categorical Boosting | iterations | int(100, 500) | 122.0 |
|  | depth | int(4, 8) | 6.000 |
|  | learning_rate | loguniform(1e-3, 1e-1) | 0.091 |
|  | l2_leaf_reg | loguniform(1e-4, 10.0) | 7.211 |
