# Supplementary figures and images for: Machine Learning–Based Prediction of In-Hospital Falls in Adult Inpatients: Retrospective Observational Multicenter Study
Source: JMIR Med Inform. 2025 Dec 4;13:e75958. doi: 10.2196/75958 (PMC12715471; doi:10.2196/75958)

**
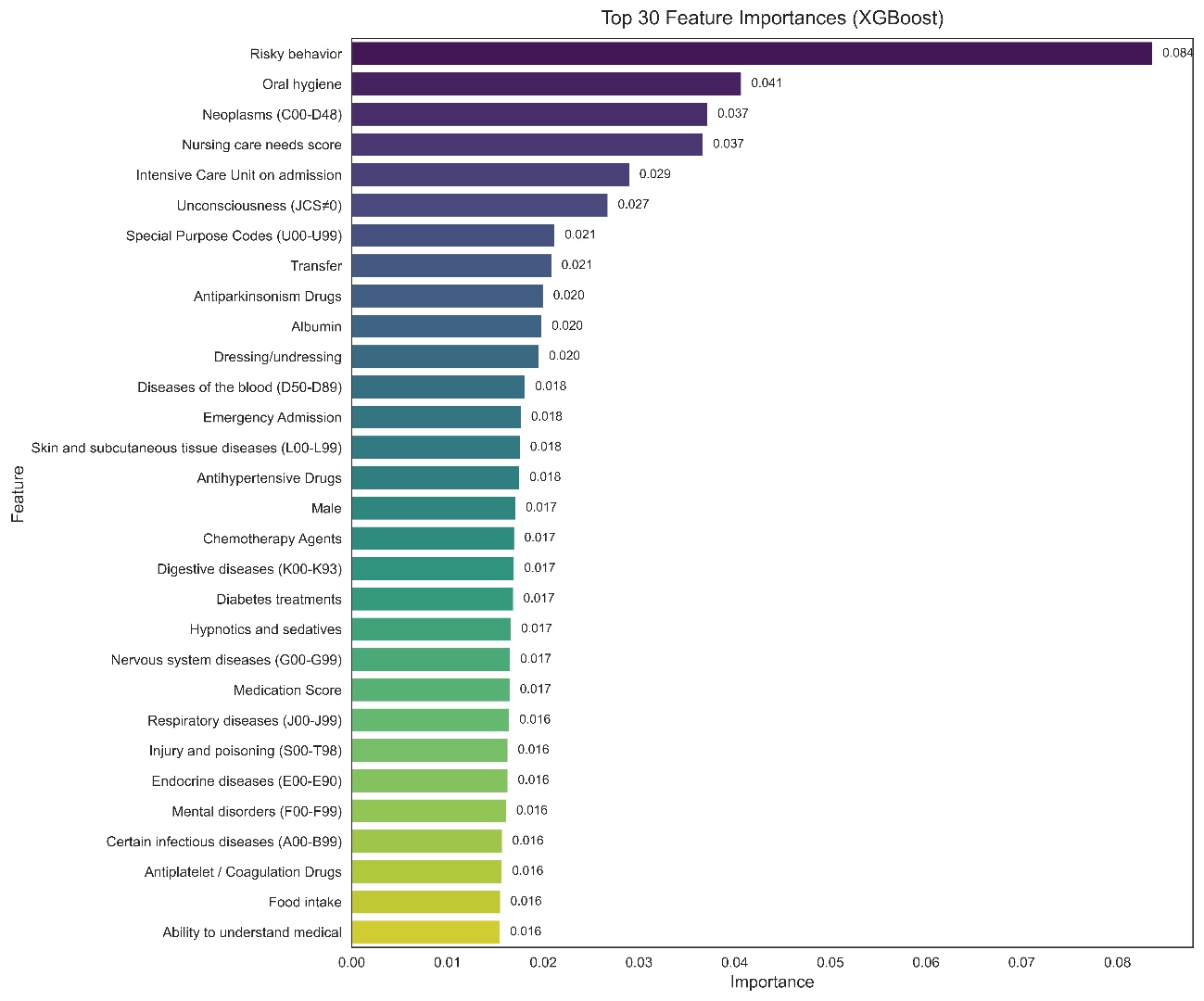
**

Note: Feature importances were calculated using an XGBoost model.

Supplement: Multimedia Appendix 2 [file medinform_v13i1e75958_app2.docx]
